# Pre-Existing Pulmonary Hypertension Impact on In-Hospital Outcomes of Cardiac Implantable Electrical Device Implantation

**DOI:** 10.1016/j.jacadv.2025.101768

**Published:** 2025-05-13

**Authors:** Gilad Margolis, Oren Mahler Hafner, Mark Kazatsker, Ariel Roguin, Eran Leshem

**Affiliations:** Division of Cardiovascular Medicine, Hillel Yaffe Medical Center, The Ruth and Bruce Rappaport Faculty of Medicine, Technion, Haifa, Israel

**Keywords:** cardiac implantable electronic devices, in-hospital outcomes, pulmonary hypertension

## Abstract

**Background:**

Pre-existing pulmonary hypertension (PH) is associated with unfavorable in-hospital outcomes in cardiac as well as noncardiac surgeries and procedures. However, its impact on cardiac implantable electronic device (CIED) implantations is not established.

**Objectives:**

The purpose of the study was to investigate the extent of pre-existing PH among patients undergoing CIED implantations and to evaluate its effect on in-hospital outcomes.

**Methods:**

Using the National Inpatient Sample database, we identified patients who were hospitalized in the United States between 2016 and 2019 and underwent CIED implantation with a pre-existing diagnosis of PH. Patients with any CIED in situ were excluded. Sociodemographic and clinical data, in-hospital procedures and outcomes, and in-hospital mortality were collected. Multivariable logistic regression models were used to identify predictors of in-hospital complications.

**Results:**

An estimated total of 718,980 patients underwent CIED implantation during the study period. Of them, 74,150 patients (10.3%) had a pre-existing PH diagnosis. Compared with non-PH patients, PH patients were older, had higher Charlson Comorbidity Index, and were more often implanted with implantable cardioverter defibrillators and cardiac resynchronization therapy devices. A higher rate of total complications was observed in PH patients (14.5% vs 9.9%; *P* < 0.001), driven mainly by respiratory complications as well as in-hospital mortality (2.3% vs 1.2%; *P* < 0.001). Multivariable analyses confirmed PH as an independent predictor for respiratory complications, total complications, and in-hospital mortality.

**Conclusions:**

Pre-existing PH in patients undergoing CIED implantation was associated with increased risk for respiratory complications as well as in-hospital mortality in a nationwide, all-comer registry.

The number of cardiac implantable electronic device (CIED) implantations is steadily increasing in the western world due to the proven improved quality of life and prolonged survival in appropriately selected patients. However, these device implantations are invasive and are associated with potential complications. Previous studies have shown that CIED implantation is associated with a periprocedural complication rate of up to 16%.^1^

Pulmonary hypertension (PH) is defined as a mean pulmonary artery pressure >20 mm Hg at rest, as measured by right heart catheterization. However, PH is most often detected noninvasively by echocardiography.^2^ PH is classified into 5 subcategories based on the underlying etiology.^3^ Pre-existing PH is a known predictor of peri-operative and periprocedural complications, including mortality, in both cardiac and noncardiac surgeries and procedures.^4^, ^5^, ^6^, ^7^, ^8^, ^9^, ^10^, ^11^ PH can potentially interfere with CIED implantation in several ways: increased right ventricular (RV) pressure, RV dilatation, or tricuspid regurgitation may cause problems with lead implantation and stabilization; venous pressure increment results in access site back bleeding with potential device pocket hematomas; conscious sedation can be cumbersome due to pre-existing respiratory problems and may result in the need for general anesthesia. While an association has been described between CIED implantation and long-term complications, specifically the development of chronic thromboembolic PH,^12^^,^^13^ the association between pre-existing PH and periprocedural outcomes of CIED implantation has not yet been evaluated.

We aimed to investigate the extent of pre-existing PH among patients undergoing CIED implantations and to evaluate its effect on in-hospital outcomes, in a contemporary, nationwide, all-comer U.S. registry.

## Methods

### Data source

The data were drawn from the National Inpatient Sample (NIS), the Healthcare Cost and Utilization Project, and the Agency for Healthcare Research and Quality.^14^^,^^15^ Access to the NIS dataset, which is available for purchase online, was granted upon completion of the Data Use Agreement Training, as required by the Agency for Healthcare Research and Quality. Once purchased, the dataset was converted to an SPSS spreadsheet, from which data retrieval was performed. The NIS database includes only deidentified data; therefore, this study was deemed exempt from institutional review by the local Human Research Committee.

The NIS is the largest collection of all-payer data on inpatient hospitalizations in the United States. The data set represents an ≈20% stratified sample of all inpatient discharges from U.S. hospitals.^16^ This information includes patient- and hospital-level factors such as demographic characteristics, primary and secondary diagnoses and procedures, comorbidities, length of stay (LOS), hospital region, hospital teaching status, hospital size, and hospitalization cost. National estimates can be calculated using the patient- and hospital-level sampling weights that are provided by the Healthcare Cost and Utilization Project.

For the purpose of this study, we obtained all data between 2016 and 2019. The International Classification of Diseases-10th Revision-Clinical Modification/Procedure Coding System (ICD-10-CM/PCS) was fully implemented for reporting diagnoses and procedures in the NIS database during the study period. Upon initiation of this study, the only ICD-10-coded NIS datasets available for analysis were of the years 2016 to 2019.

For each index hospitalization, the database provides a principal discharge diagnosis and a maximum of 39 additional diagnoses, in addition to a maximum of 25 procedures.

### Study population and variables

We identified within the NIS database patients aged ≥18 years who underwent CIED implantation during the study period using ICD-10-PCS codes: 02HKXJZ, 02H6XJZ, 0JH6X4Z, 0JH6X5Z, 0JH6X6Z, 0JH8X4Z, 0JH8X5Z, and 0JH8X6Z for permanent pacemakers; 02HKXKZ, 0JH6X8Z, and 0JH8X8Z for implantable cardioverter-defibrillators; and 0JH6X7Z, 0JH6X9Z, 02H4XJZ, 02H4XNZ, 0JH8X7Z, and 0JH8X9Z for cardiac resynchronization therapy (CRT) device implantation procedures. Patients with a pre-existing CIED in situ were excluded using ICD-10-CM codes Z950, Z450XX, and Z95810. Pre-existing PH was identified using ICD-10-CM codes I270 and I272X.

The following patient demographics were collected from the database: age, sex, and race. ICD-10-CM codes (Supplemental Table 1) were used to identify different comorbidities including hypertension, congestive heart failure, diabetes mellitus, chronic kidney disease, chronic pulmonary disease, obesity, peripheral vascular disease, and obstructive sleep apnea, as well as in-hospital diagnoses and procedures, including syncope, cardiogenic shock, RV failure, acute myocardial infarction, and temporary pacemaker. Electrocardiography findings and other hospital characteristics were identified as well (provided in detail in Table 1).

**Table 1:** Demographics, Risk Factors, and Hospital Characteristics

	PH (NE = 74,150)	Non-PH (NE = 644,830)	*P* Value
Age (y)	74 ± 13	73 ± 13	<0.001
Female	37,285 (50.3)	273,270 (42.4)	<0.001
Ethnicity			
White	52,810 (73.5)	479,250 (76.6)	<0.001
Black	10,000 (13.9)	62,320 (10)	<0.001
Hispanic	5,205 (7.2)	49,365 (7.9)	<0.001
Asian	1,650 (2.3)	15,885 (2.5)	<0.001
Hypertension	15,230 (20.5)	248,045 (38.5)	<0.001
Congestive heart failure	56,920 (76.8)	306,485 (47.5)	<0.001
Diabetes mellitus	26,208 (35.4)	202,735 (31.4)	<0.001
Chronic kidney disease	33,945 (45.8)	187,035 (29)	<0.001
Chronic pulmonary disease	21,120 (28.5)	116,750 (18.1)	<0.001
Obesity	16,925 (22.8)	110,985 (17.2)	<0.001
Peripheral vascular disease	8,795 (11.9)	56,295 (8.7)	<0.001
OSA	14,605 (19.7)	84,705 (13.1)	<0.001
Smoking	26,170 (35.3)	233,080 (36.1)	<0.001
Alcohol dependence	965 (1.3)	8,100 (1.3)	0.295
In-hospital diagnoses and procedures			
Syncope	3,915 (5.3)	55,525 (8.6)	<0.001
Cardiogenic shock	5,335 (7.2)	24,710 (3.8)	<0.001
Right ventricular failure	1,585 (2.1)	1,440 (0.2)	<0.001
Acute myocardial infarction	11,305 (15.2)	93,010 (14.4)	<0.001
Temporary pacemaker	7,725 (10.4)	61,640 (9.6)	<0.001
ECG findings			
Atrial fibrillation/flutter	44,765 (60.4)	276,635 (42.9)	<0.001
Ventricular fibrillation/tachycardia	13,880 (18.7)	105,985 (16.4)	<0.001
Conduction disorder in prior ECG	22,745 (30.7)	190,475 (29.5)	<0.001
Complete AV block	22,255 (30)	195,655 (30.3)	0.065
Sick sinus syndrome	25,130 (33.9)	224,800 (34.9)	<0.001
Cardiac arrest	4,340 (5.9)	40,745 (6.3)	<0.001
Deyo-CCI			<0.001
0	4,990 (6.7)	129,310 (20.1)	
1-2	29,780 (40.2)	284,605 (44.1)	
≥3	39,380 (53.1)	230,915 (35.8)	
CIED type			
PPM	52,660 (71)	488,275 (75.7)	<0.001
ICD	19,485 (26.3)	145,540 (22.6)	<0.001
CRT	12,785 (17.2)	74,385 (11.5)	<0.001
Income percentile			<0.001
0-25	19,955 (27.4)	170,005 (26.8)	
26-50	19,005 (26.1)	166,655 (26.3)	
51-75	18,340 (25.1)	158,835 (25)	
76-100	15,630 (21.4)	138,915 (21.9)	
Hospital status			<0.001
Rural	2,695 (3.6)	30,460 (4.7)	
Nonteaching	13,335 (18)	131,495 (20.4)	
Teaching	58,120 (78.4)	482,875 (74.9)	
Hospital region			<0.001
Northeast	14,665 (19.8)	127,770 (19.8)	
Midwest	18,145 (24.5)	142,525 (22.1)	
South	26,725 (36)	251,555 (39)	
West	14,615 (19.7)	122,980 (19.1)	

Values are mean ± SD or n (%). Variables with missing data (NE, %): gender (80, <0.1%), income percentile (11,640, 1.6%).

AV = atrioventricular; CIED = cardiac implantable electronic device; CRT = cardiac resynchronization therapy; Deyo-CCI = Deyo-Charlson Comorbidity Index; ECG = electrocardiography; ICD = implantable cardioverter defibrillator; NE = national estimate of hospitalizations (see main text); OSA = obstructive sleep apnea; PH = pulmonary hypertension; PPM = permanent pacemaker.

The Charlson–Deyo Comorbidity Index (a modification of the Charlson Comorbidity Index, containing 19 comorbidity conditions) was calculated with additional comorbidities identified from the database.^17^^,^^18^ Detailed information on the Charlson–Deyo comorbidity index is provided in Supplemental Table 2. Higher Charlson–Deyo comorbidity index scores indicate a greater burden of comorbid diseases and are associated with mortality 1 year after admission. The index has been used extensively in studies from administrative databases, with proven validity in predicting short- and long-term outcomes.^18^, ^19^, ^20^

The primary outcome in this study was in-hospital complications including in-hospital mortality as well as hospital LOS and total charges. In-hospital complications were identified using ICD-10-CM/PCS codes (Supplemental Table 1): hemopericardium, tamponade, acute pericarditis, pneumothorax/hemothorax, chest tube insertion, postoperative respiratory failure, pleural effusion, pocket hematoma, lead dislodgment, postprocedural fever, and acute access deep vein thrombosis.

### Statistical analysis

Frequencies and proportions of the different demographic, clinical, and hospital-related variables were calculated and weighted to reflect national estimates using discharge sample weights provided by the NIS. These estimates were compared according to PH/non-PH grouping using the Pearson chi-square test and independent-samples t-test for categorical variables and continuous variables, respectively.

Multivariable binary logistic regression models were generated for each of the in-hospital complications that showed significant differences between patients with and without pre-existing PH in univariable analysis. Candidate variables for these models included patient characteristics, the Charlson–Deyo Comorbidity Index, and hospital-level factors. In our final multivariable regression models, which were performed in enter mode, we incorporated all candidate variables that showed an association with in-hospital complications in the univariable analysis. For all analyses, we used SPSS software version 23 (IBM Corp). A *P* value <0.05 was considered statistically significant.

## Results

### Study cohort

A total of 143,796 CIED implantation procedures were performed across the United States during the study period. After implementing the weighting method, these represented an estimated total of 718,980 hospitalizations in which CIED was implanted. The majority of patients (56.8%) were men, and the mean age was 73 ± 13 years.

### Patients' characteristics according to the presence of PH

Baseline characteristics of the study population in the PH/non-PH groups are presented in detail in Table 1. A total of 74,150 patients (10.3%) had pre-existing PH. Older age and female sex were more prevalent in the PH group. Patients with pre-existing PH were more likely to have a history of congestive heart failure, chronic pulmonary disease, obesity, and obstructive sleep apnea and had a higher Charlson–Deyo Comorbidity Index.

In addition, patients in the PH group were more likely to have a diagnosis of cardiogenic shock, RV failure, acute myocardial infarction, and atrial fibrillation, as well as ventricular fibrillation. Pre-existing PH patients had higher rates of temporary cardiac pacing as well as implantable cardioverter-defibrillator and CRT device implantations and lower rates of permanent pacemaker implantations compared with non-PH patients.

### In-hospital course and outcomes

Of the total population that underwent CIED implantation, 10.3% had an in-hospital complication. Compared with those without PH, patients with pre-existing PH had a higher in-hospital mortality (2.3% vs 1.2%; *P* < 0.001) and unadjusted rate of any in-hospital complications (14.5% vs 9.9%; *P* < 0.001) (Central Illustration). The higher complication rate in the PH group was driven by increased rate of chest tube insertion (5.3% vs 3%; *P* < 0.001), pneumo/hemothorax (2.4% vs 2.1%; *P* < 0.001), postoperative respiratory failure (1.3% vs 0.6%; *P* < 0.001), pleural effusion (6.3% vs 3.2%; *P* < 0.001), pocket hematoma (0.7% vs 0.5%; *P* < 0.001), and acute access deep vein thrombosis (0.9% vs 0.7%; *P* < 0.001) (Table 2). Conversely, PH patients had a lower rate of lead dislodgment (1.1% vs 1.3%; *P* < 0.001) (Table 2). Stratification by RV failure showed that pre-existing PH patients with concomitant RV failure had a significantly higher unadjusted rate of any in-hospital complications (23.3% vs 14.3%; *P* < 0.001) and in-hospital mortality (4.7% vs 2.2%; *P* < 0.001) compared to those without RV failure (Supplemental Table 3).

**Central Illustration:**
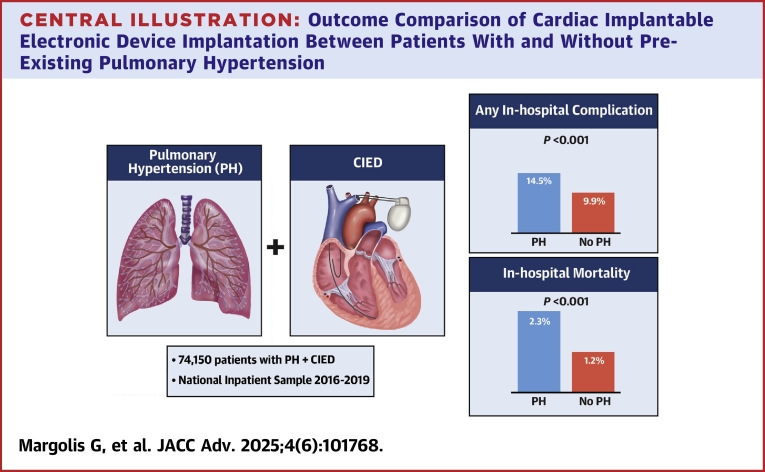
Outcome Comparison of Cardiac Implantable Electronic Device Implantation Between Patients With and Without Pre-Existing Pulmonary Hypertension CIED = cardiac implantable electronic device; PH = pulmonary hypertension.

**Table 2:** In-Hospital Outcomes After CIED Implantation

	PH (NE = 74,150)	Non-PH (NE = 644,830)	Unadjusted OR (95% CI)	*P* Value
Any in-hospital complications	10,760 (14.5)	63,615 (9.9)	1.55 (1.52-1.59)	<0.001
Pericardial complications	439 (0.6)	3,845 (0.6)	0.97 (0.88-1.08)	0.58
Pneumothorax/hemothorax	1,780 (2.4)	13,485 (2.1)	1.15 (1.10-1.21)	<0.001
Chest tube insertion	3,965 (5.3)	19,265 (3)	1.83 (1.77-1.9)	<0.001
Postoperative respiratory failure	940 (1.3)	4,170 (0.6)	1.97 (1.84-2.12)	<0.001
Pleural effusion	4,675 (6.3)	20,700 (3.2)	2.03 (1.96-2.1)	<0.001
Pocket hematoma	500 (0.7)	3,165 (0.5)	1.38 (1.25-1.51)	<0.001
Lead dislodgment	815 (1.1)	8,065 (1.3)	0.88 (0.82-0.94)	<0.001
Postprocedural fever	480 (0.6)	4,080 (0.6)	1.02 (0.93-1.13)	0.635
Acute access DVT	660 (0.9)	4,545 (0.7)	1.27 (1.17-1.37)	<0.001
In-hospital mortality	1,685 (2.3)	7,640 (1.2)	1.94 (1.84-2.05)	<0.001
Length of stay	8.8 ± 8.5	6.1 ± 7.3	NA	<0.001
Total hospitalization charges	197,398 ± 202,457	149,949 ± 168,070	NA	<0.001

Values are n (%) or mean ± SD. Variables with missing data (NE, %): in-hospital mortality (340, <0.1%), total hospital charges (5,540, 0.8%), and length of stay (30, 0.1%).

Pericardial complications consist of hemopericardium, tamponade, and acute pericarditis.

CIED = cardiac implantable electronic device; DVT = deep vein thrombosis; NA = not applicable; NE = national estimate of hospitalizations (see main text); PH = pulmonary hypertension.

Subgroup analyses showed that pre-existing PH patients aged over 60 years were more likely to have lead dislodgment (unadjusted OR: 1.13; 95% CI: 1.05-1.21) but less likely to have pocket hematoma (unadjusted OR: 0.71; 95% CI: 0.65-0.78). Female patients with pre-existing PH were prone to lead dislodgment (unadjusted OR: 1.24; 95% CI: 1.12-1.37), but less likely to experience pneumo/hemothorax (unadjusted OR: 0.78; 95% CI: 0.73-0.83). Pre-existing PH patients of White ethnicity were more likely to have lead dislodgment (unadjusted OR: 1.12; 95% CI: 1.04-1.21).

The average LOS for the entire study population was 6.4 ± 7.5 days. Patients with pre-existing PH had a longer LOS in the hospital compared with those without PH (8.8 ± 8.5 days vs 6.1 ± 7.3 days; *P* < 0.001). The mean total hospital charges for the whole study population were 154,829 ± 172,529 U.S. dollars, and these were significantly higher in patients with PH compared to non-PH patients (197,398 ± 202,457 vs 149,949 ± 168,070 U.S. dollars; *P* < 0.001) (Table 2).

### Predictors of in-hospital complications

**Figure 1:**
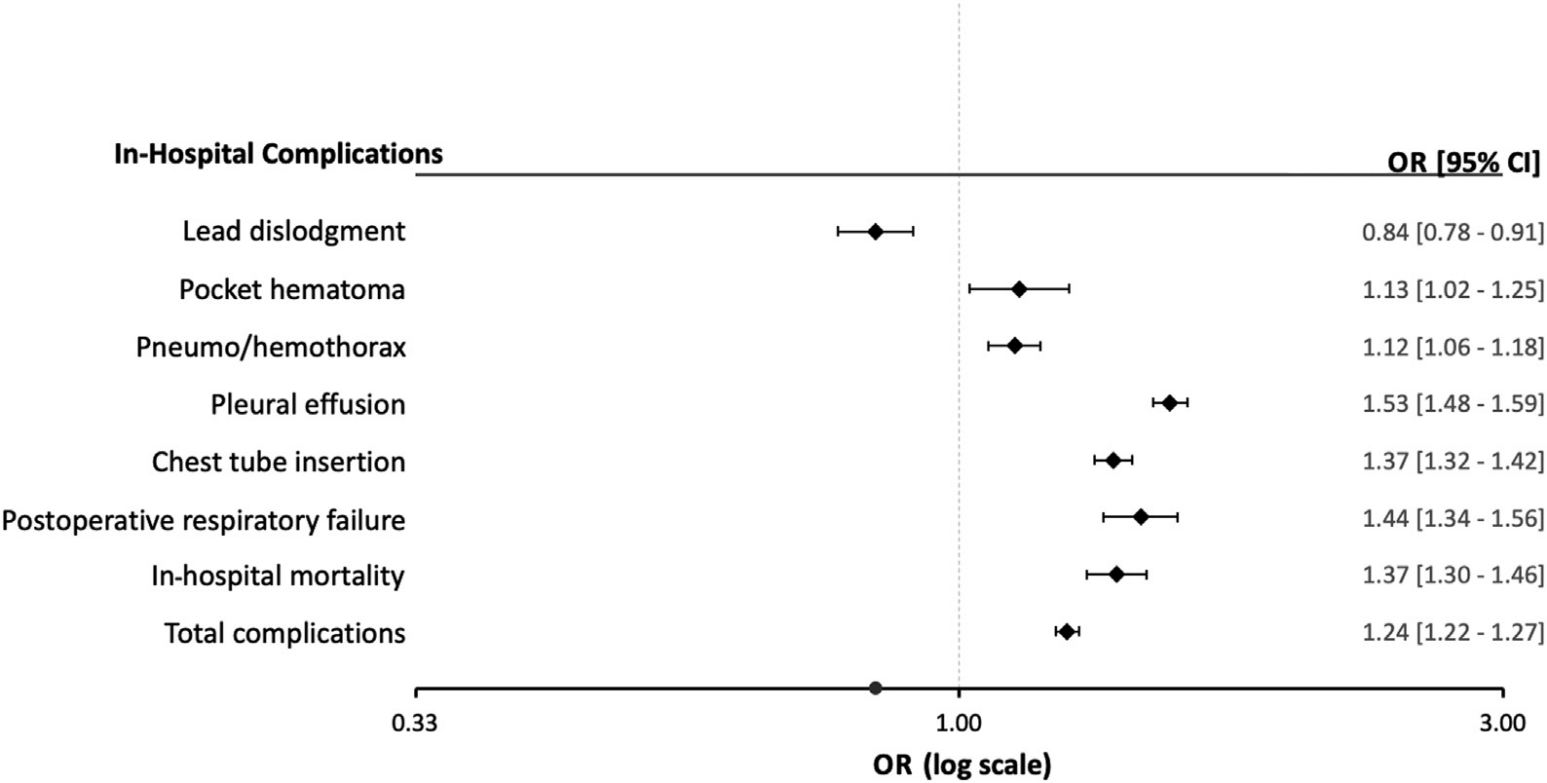
Adjusted ORs for In-Hospital Complications by Pulmonary Hypertension Presence The complication risk for patients with pre-existing PH was adjusted for patient- and hospital-level variables that showed an association with in-hospital complications in the univariable analysis. The comprehensive multivariate analyses, encompassing all variables, are presented in Supplemental Tables 4 to 11. PH = pulmonary hypertension.

In multivariate analyses adjusted for the significantly associated baseline characteristics of in-hospital complications, PH emerged as an independent predictor of chest tube insertion (OR: 1.36 [95% CI: 1.31-1.41]; *P* < 0.001), pneumo/hemothorax (OR: 1.13 [95% CI: 1.07-1.19]; *P* < 0.001), postoperative respiratory failure (OR: 1.44 [95% CI: 1.33-1.55]; *P* < 0.001), pleural effusion (OR: 1.52 [95% CI: 1.47-1.57]; *P* < 0.001), pocket hematoma (OR: 1.15 [95% CI: 1.04-1.27]; *P* = 0.005), in-hospital mortality (OR: 1.36 [95% CI: 1.28-1.44]; *P* < 0.001), and any complications during hospitalization (OR: 1.24 [95% CI: 1.20-1.27]; *P* < 0.001) (Figure 1, Supplemental Tables 5 to 11). In contrast, this analysis showed that PH was significantly associated with a lower risk for lead dislodgment (OR: 0.84 [95% CI: 0.78-0.90]; *P* < 0.001) (Figure 1, Supplemental Table 4).

## Discussion

Using data from the NIS, the largest all-payer inpatient database in the United States, we analyzed a weighted total of 718,980 patients who underwent CIED implantation between 2016 and 2019. An estimated total of 74,150 patients (10.3%) had pre-existing PH. Our analysis showed that pre-existing PH is associated with increased in-hospital mortality, longer hospitalization duration, higher costs, and procedural-related complications, driven mainly by respiratory complications.

Pre-existing PH patients had a higher periprocedural mortality rate (2.3% vs 1.2%) compared with non-PH patients. Pre-existing PH patients with concomitant RV failure were prone to even higher in-hospital mortality (unadjusted rate 4.7%) (Supplemental Table 3) and complications, making them potentially a very high-risk group. However, the relatively small number of RV failure patients precludes performing multivariate analyses to assess the independent effect of RV failure on outcomes in pre-existing PH patients. In previous studies, pre-existing PH was also associated with increased mortality risk in both cardiac and noncardiac surgeries and procedures.^4^^,^^5^^,^^8^, ^9^, ^10^, ^11^ In patients undergoing transvenous and surgical aortic valve replacement, pre-existing PH was associated with decreased short- and long-term survival.^4^^,^^5^^,^^21^, ^22^, ^23^ Pre-existing PH was also identified as a risk factor for increased long-term mortality after mitral valve transcatheter repair and surgery.^6^^,^^7^^,^^24^, ^25^, ^26^ In noncardiac surgery, underlying PH is a predictor for worse peri-operative outcomes,^8^^,^^9^ including orthopedic,^27^ renal,^11^ and lung surgery.^10^

Similar to our findings, respiratory complications have been reported as the most frequent peri-operative complication in both cardiac^5^ and noncardiac surgeries^9^^,^^28^ among patients with pre-existing PH. Our analysis identified pre-existing PH as a significant predictor for pneumo/hemothorax. Notably, PH patients in the current study were more likely to undergo temporary pacing and receive more complex CIEDs such as CRT devices, which usually require more venous access punctures, thereby elevating the risk for pneumo/hemothorax. PH patients were also more likely to experience postoperative respiratory failure in the current analysis. PH patients, some with compromised pulmonary reserves at baseline, may develop hypoventilation due to residual analgesia or sedation.^27^ The higher rates of pleural effusions observed in PH patients compared with non-PH patients may be a manifestation of congestive heart failure, which was more frequent among PH patients in our study.

Our analysis shows that pre-existing PH emerged as a significant predictor for pocket hematoma in multivariable analysis. Elevated venous pressure can result in increased back bleeding at access sites and these may lead to higher rates of device pocket hematomas. PH patients were more likely to have an atrial fibrillation diagnosis. Although we lack specific data, it is reasonable to assume they were also more likely to receive chronic anticoagulation therapy, which may additionally explain their higher bleeding risk. Interestingly, pre-existing PH was associated with a lower risk for lead dislodgment. The lower risk of lead dislodgment observed in the PH group may be explained by operator awareness of the PH diagnosis or intraprocedural difficulty with initial lead fixation due to elevated RV pressure, which could lead to more careful lead fixation during implantation. However, as no detailed intraprocedural data exists, this assumption remains speculative.

### Study limitations

Several limitations need to be acknowledged. First, the NIS database is a retrospective administrative database that contains discharge-level records and, as such, is susceptible to coding errors, as well as data retrieval errors due to missing or mistyped codes. We cannot exclude the possibility that some patients may have been mislabeled with a pre-existing PH diagnosis because the NIS database does not provide information regarding the evaluations, including echocardiography and/or right heart catheterization that patients underwent before being diagnosed with PH. The database does not allow for distinction between newly diagnosed and prior PH. Patients with reversible forms of PH could not be identified. For instance, patients with decompensated heart failure and secondary PH are likely to fare worse than those who were diuresed and had compensated heart failure. However, the dataset does not allow for such detailed analysis. Furthermore, we were unable to reliably identify PH subgroups, as the database lacks the granularity required to differentiate between them. Nearly half the study cohort was diagnosed with unspecified PH (ICD-10-CM code I27.20), reflecting potential misclassification bias. In addition, PH severity measures are not provided in the dataset. It is likely that, compared with mild PH patients, those with severe PH had worse in-hospital outcomes. However, it was not possible to evaluate the specific effects of different PH types or severity on patients' outcomes. Lack of patient identifiers in the NIS database prevented us from using other outcome variables and death measures such as 30-day deaths, and only events that occurred in the same index hospitalization were captured. The NIS database also does not include detailed information about patients' clinical characteristics, medication, blood tests, and so on. Therefore, we cannot rule out residual confounding of the associations observed. These limitations are counterbalanced by the real-world, nationwide nature of the data, lack of selection bias, and absence of reporting bias introduced by selective publication of results from specialized centers.

## Conclusions

Pre-existing PH is present in 1 out of every 10 patients undergoing CIED implantation in the United States and is associated with an increased risk for respiratory complications as well as in-hospital mortality. These findings should be interpreted with caution and viewed as hypothesis-generating due to the retrospective study design. Until future prospective studies confirm these observations, a careful preprocedural risk-benefit assessment of the necessity for CIED implantation, as well as the complexity of the device and procedure, would be prudent for this high-risk patient group.

Perspectives **COMPETENCY IN PATIENT CARE AND PROCEDURAL SKILLS:** Pre-existing PH was observed in 1 out of every 10 patients undergoing CIED implantation in the United States. This condition was associated with worse periprocedural outcomes.

**TRANSLATIONAL OUTLOOK:** Future prospective studies are necessary to validate these observations. In the meantime, it would be prudent to assess both the necessity and complexity of the device before implantation in patients with pre-existing PH.

## Funding support and author disclosures

The authors have reported that they have no relationships relevant to the contents of this paper to disclose.
