# Supplementary material for: Pre-Existing Pulmonary Hypertension Impact on In-Hospital Outcomes of Cardiac Implantable Electrical Device Implantation
Source: JACC Adv. 2025 May 13;4(6):101768. doi: 10.1016/j.jacadv.2025.101768 (PMC12142518; doi:10.1016/j.jacadv.2025.101768)
Supplement: Supplementary data [file mmc1.docx]

Supplemental Table 1: ICD-10 CM/PCS codes used in data extraction

| ICD-10 CM/PCS codes | Condition/Procedure |
| --- | --- |
| Codes for Inclusion criteria and for Preexisting Pulmonary hypertension patients | |
| 02HKXJZ, 02H6XJZ , 0JH6X4Z, 0JH6X5Z, 0JH6X6Z, 0JH8X4Z, 0JH8X5Z, 0JH8X6Z | permanent pacemakers (PPM) |
| 02HKXKZ, 0JH6X8Z, 0JH8X8Z | implantable cardioverter-defibrillators (ICD) |
| 0JH6X7Z, 0JH6X9Z, 02H4XJZ, [02H4XNZ](https://www.icd10data.com/ICD10PCS/Codes/0/2/H/4/02H40NZ), 0JH8X7Z, 0JH8X9Z | cardiac resynchronization therapy (CRT) |
| I270, I272X | Pulmonary hypertension |
| Codes for Exclusion criteria | |
| Z950, Z95810, Z450XX | Cardiac implantable electronic device in-situ |
| Codes for in-hospital diagnoses and procedures | |
| R55, I459 | Syncope |
| R570 | Cardiogenic shock |
| I50.81 I50.810 I50.811 I50.812 I50.813 I50.814, I27.81 | Right ventricular failure |
| I21X, I22X, I252 | Acute myocardial infarction |
| 5A12X3Z | Temporary pacemaker |
| Codes for ECG findings | |
| [I48.3](https://www.icd10data.com/ICD10CM/Codes/I00-I99/I30-I5A/I48-/I48.3)  [I48.4](https://www.icd10data.com/ICD10CM/Codes/I00-I99/I30-I5A/I48-/I48.4)  [I48.9](https://www.icd10data.com/ICD10CM/Codes/I00-I99/I30-I5A/I48-/I48.9)  [I48.91](https://www.icd10data.com/ICD10CM/Codes/I00-I99/I30-I5A/I48-/I48.91)  [I48.92](https://www.icd10data.com/ICD10CM/Codes/I00-I99/I30-I5A/I48-/I48.92) | Atrial flutter |
| [I48](https://www.icd10data.com/ICD10CM/Codes/I00-I99/I30-I5A/I48-/I48)  [I48.0](https://www.icd10data.com/ICD10CM/Codes/I00-I99/I30-I5A/I48-/I48.0)  [I48.1](https://www.icd10data.com/ICD10CM/Codes/I00-I99/I30-I5A/I48-/I48.1)  [I48.11](https://www.icd10data.com/ICD10CM/Codes/I00-I99/I30-I5A/I48-/I48.11)  [I48.19](https://www.icd10data.com/ICD10CM/Codes/I00-I99/I30-I5A/I48-/I48.19) [I48.2](https://www.icd10data.com/ICD10CM/Codes/I00-I99/I30-I5A/I48-/I48.2)  [I48.20](https://www.icd10data.com/ICD10CM/Codes/I00-I99/I30-I5A/I48-/I48.20)  [I48.21](https://www.icd10data.com/ICD10CM/Codes/I00-I99/I30-I5A/I48-/I48.21) | Atrial fibrillation |
| I472 I4720 I4729 | Ventricular tachycardia |
| I4901 I490 | Ventricular fibrillation |
| I440, I441, I447, I450, I451, I4510, I444, I445, I452, I453, I454A | Conduction disorder in prior ECG |
| I442 | Complete AV block |
| I495 | Sick sinus syndrome |
| I46x | Cardiac arrest |
| Codes for baseline characteristics and comorbidities not included in Deyo-CCI | |
| I10 | Hypertension |
| E66X, E66.09, Z68.4, Z68.3 | Obesity |
| Z87891, F17210, F17200 | Smoking |
| F102XXX | Alcohol dependence |
| G4730, G4733 | obstructive sleep apnea |
| Codes for in-hospital complications | |
| I312 | Hemopericardium |
| I314 | Tamponade |
| I30X | Acute pericarditis |
| J938X, J939, J942, J95811 | Pneumothorax/hemothorax |
| 0W99XXX, 0W9BXXX | Chest tube insertion |
| J9582X | Post-operative respiratory failure |
| J918, J91, J90 | Pleural effusion |
| T82897A | Pocket hematoma |
| T8212XX | Lead dislodgement |
| R509, R5082 | Post-procedural fever |
| T8172XX, I8229X, I82AX, I82BX, I826XX | Acute access deep vein thrombosis |

AV= atrioventricular, ICD-10-CM/PCS International Classification of Diseases, 10^th^ Revision, Clinical Modification/ Procedure Coding System

Supplemental Table 2: ICD-10 CM Codes for conditions incorporated in Deyo-CCI, and scoring system used to compute Deyo-CCI scores

| ICD-10 CM codes | Condition | Score |
| --- | --- | --- |
| I21.x, I22.x, I25.2 | Myocardial infarction | 1 |
| I11.0, I13.0, I13.2, I25.5, I42.0, I42.5-I42.9, I43.x, I50.x, P29.0 | Congestive heart failure | 1 |
| I70.x, I71.x, I73.1, I73.8, I73.9, I77.1, I79.0, I79.1, I79.8, K55.1, K55.8, K55.9, Z95.8, Z95.9 | Peripheral vascular disease | 1 |
| G45.x, G46.x, H34.0x, H34.1x, H34.2x, I60.x-I68.x | Cerebrovascular disease | 1 |
| F01.x-F03.x, F04, F05, F06.1, F06.8, G13.2, G13.8, G30.x, G31.0x, G31.1, G31.2, G91.4, G94, R41.81, R54 | Dementia | 1 |
| J40.x-J47.x, J60.x-J67.x, J68.4, J70.1, J70.3 | Chronic pulmonary disease | 1 |
| M05.x, M06.x, M31.5, M32.x-M34.x, M35.1, M35.3, M36.0 | Rheumatologic disease | 1 |
| K25.x-K28.x | Peptic ulcer disease | 1 |
| B18.x, K70.0-K70.3, K70.9, K71.3-K71.5, K71.7, K73.x, K74.x, K76.0, K76.2-K76.4, K76.8, K76.9, Z94.4 | Mild liver disease | 1 |
| Main codes: E08, E09, E10, E11, E13.  Relevant subcodes: E**.0x, E**.1x, E**.6x, E**.8x, E**.9x | Diabetes without chronic complications | 1 |
| I12.9, I13.0, I13.10, N03.x, N05.x, N18.1-N18.4, N18.9, Z94.0 | Renal disease, mild or moderate | 1 |
| Main codes: E08, E09, E10, E11, E13.  Relevant subcodes: E**.2x, E**.3x, E**.4x, E**.5x | Diabetes with chronic complications | 2 |
| G04.1, G11.4, G80.0, G80.1, G80.2, G81.x, G82.x, G83.x | Hemiplegia or paraplegia | 2 |
| C0x.x, C1x.x, C2x.x, C30.x-C34.x, C37.x-C41.x, C43.x, C45.x-C58.x, C60.x-C63.x,C76.x, C80.1, C81.x-C85.x, C88.x, C9x.x | Any malignancy including leukemia and lymphoma except malignant nonmelanoma of the skin | 2 |
| I85.0x, I86.4, K70.4x, K71.1x, K72.1x, K72.9x, K76.5, K76.6, K76.7 | Moderate or severe liver disease | 3 |
| I12.0, I13.11, I13.2, N18.5, N18.6, N19.x, N25.0, Z49.x, Z99.2 | Renal disease, severe | 3 |
| B20.x | HIV infection | 3 |
| C77.x-C79.x, C80.0, C80.2 | Metastatic solid tumor | 6 |
| B37.x, C53.x, B38.x, B45.x, A07.2, B25.x, G93.4x, B00, B39.x, A07.3, C46.x, C81-96, A31.x, A15-19, B59, Z87.01, A81.2, A02.1, B58.x, R64 | Acquired Immunodeficiency syndrome (AIDS) | 6 |

Deyo- CCI= Deyo- Charlston Comorbidity Index, ICD-10-CM =International Classification of Diseases, 10^th^ Revision, Clinical Modification.

Supplemental Table 3. In-Hospital Outcomes following CIED Implantation in Patients with/without PH, Stratified by RV failure

| **Variable** | **PH**  **(NE=74150)** | | **Non-PH**  **(NE=644830)** | | **P value** |
| --- | --- | --- | --- | --- | --- |
|  | **RV failure**  **(NE=1585)** | **No-RV failure**  **(NE=72565)** | **RV failure**  **(NE=1440)** | **No-RV failure**  **(NE=643390)** |  |
| Any in-hospital complications | 370 (23.3) | 10390 (14.3) | 245 (17.0) | 63370 (9.9) | <0.001 |
| Pneumothorax/hemothorax | 20 (1.3) | 1760 (2.4) | 30 (2.1%) | 13455 (2.1) | 0.003 |
| Pleural effusion | 175 (11.0) | 4500 (6.2) | 95 (6.6) | 20605 (3.2) | <0.001 |
| Chest tube insertion | 150 (9.5) | 3815 (5.3) | 70 (4.9) | 19195 (3.0) | <0.001 |
| Post-operative respiratory failure | 20 (1.3) | 920 (1.3%) | 20 (1.4) | 4150 (0.6%) | 0.986 |
| In-hospital mortality | 75 (4.7) | 1610 (2.2) | 60 (4.2) | 7580 (1.2) | <0.001 |
| Length of stay | 10.9 ± 10.1 | 8.8 ± 8.5 | 11.4 ± 11.6 | 6.1 ± 7.3 | <0.001 |

Pericardial complications, lead dislodgement, pocket hematoma, post-procedural fever and acute access DVT rates were ≤10 in some patient category cells and therefore could not be reported per Agency for Healthcare Research and Quality (AHRQ) guidelines for anonymizing data. DVT= deep vein thrombosis; NE= national estimate of hospitalizations (see main text); OR= odds ratio; PH= pulmonary hypertension; RV= right ventricle. Categorical variables are reported as incidence and frequencies n(%) while continuous variables are summarized as mean±SD.

P values reported in this table are for comparisons between preexisting PH patients with/without RV failure.

Supplemental Table 4: Predictors for lead dislodgement

| p value | **95 CI** | OR | **Correlates** |
| --- | --- | --- | --- |
| 0.006 | 0.99-0.99 | 0.99 | Age |
| <0.001 | 1.06-1.16 | 1.11 | Female |
| <0.001 | 1.09-1.21 | 1.15 | White ethnicity |
| 0.58 | 0.96-1.07 | 1.01 | Hypertension |
| 0.022 | 1.01-1.14 | 1.07 | CHF |
| 0.032 | 1.01-1.13 | 1.06 | CPD |
| <0.001 | 1.28-1.43 | 1.35 | Obesity |
| 0.21 | 0.88-1.03 | 0.95 | PVD |
| 0.005 | 1.03-1.16 | 1.09 | OSA |
| <0.001 | 0.71-0.84 | 0.77 | Syncope |
| <0.001 | 0.70-0.90 | 0.79 | Cardiogenic shock |
| <0.001 | 0..77-0.88 | 0.82 | MI |
| <0.001 | 0.74-0.85 | 0.79 | VT/VF |
| <0.001 | 0.87-0.96 | 0.91 | Disorder in prior ECG |
| 0.001 | 0.88-0.97 | 0.92 | Sick sunus syndrome |
| <0.001 | 0.67-0.82 | 0.74 | Cardiac arrest |
| <0.001 | 1.23-1.41 | 1.32 | CRT |
| 0.001 | 0.83-0.95 | 0.89 | PPM |
| <0.001 | 0.89-0.96 | 0.93 | Deyo-CCI |
| 0.77 | 0.96-1.05 | 1.01 | Hospital region- south/west |
| <0.001 | 0.78-0.91 | 0.844 | PH |

PH = pulmonary hypertension; CHF = congestive heart failure; CPD = chronic pulmonary disease; Deyo- CCI= Deyo- Charlson Comorbidity Index; PVD = peripheral vascular disease; OSA = obstructive sleep apnea; MI = myocardial infarction; VF/VT= ventricular fibrillation/tachycardia; CRT= cardiac resynchronization therapy; PPM= permanent pacemaker.

Supplemental Table 5: Predictors for in hospital mortality

| p value | **95 CI** | OR | **Correlates** |
| --- | --- | --- | --- |
| <0.001 | 1.01-1.01 | 1.01 | Age |
| 0.941 | 0.95-1.05 | 0.99 | Female |
| <0.001 | 0.83-0.92 | 0.88 | White ethnicity |
| <0.001 | 0.59-0.67 | 0.63 | Hypertension |
| <0.001 | 1.24-1.40 | 1.32 | CHF |
| <0.001 | 0.85-0.94 | 0.89 | Diabetes mellitus |
| <0.001 | 1.06-1.17 | 1.11 | CKD |
| 0.005 | 1.02-1.14 | 1.08 | CPD |
| <0.001 | 0.65-0.74 | 0.69 | Obesity |
| <0.001 | 1.11-1.27 | 1.19 | PVD |
| <0.001 | 0.62-0.73 | 0.67 | OSA |
| <0.001 | 0.49-0.62 | 0.55 | Syncope |
| <0.001 | 6.39-7.12 | 6.74 | Cardiogenic shock |
| <0.001 | 1.33-1.99 | 1.63 | RV failure |
| <0.001 | 0.83-0.94 | 0.88 | MI |
| 0.44 | 0.94-1.03 | 0.98 | AF/AFL |
| <0.001 | 2.11-2.37 | 2.24 | VT/VF |
| <0.001 | 0.51-0.57 | 0.54 | Disorder in prior ECG |
| 0.017 | 0.89-0.99 | 0.94 | Complete AV block |
| <0.001 | 0.62-0.69 | 0.66 | Sick sunus syndrome |
| <0.001 | 4.88-5.44 | 5.15 | Cardiac arrest |
| <0.001 | 1.68-1.88 | 1.78 | Temporary pacemaker |
| <0.001 | 0.32-0.42 | 0.36 | ICD |
| <0.001 | 1.66-2.15 | 1.89 | PPM |
| <0.001 | 1.88-2.09 | 1.98 | Deyo- CCI |
| <0.001 | 0.61-0.68 | 0.65 | History of smoking |
| 0.001 | 0.55-0.85 | 0.69 | Alcohol dependency |
| 0.03 | 1.03-1.15 | 1.09 | Teaching hospital |
| <0.001 | 0.93-0.96 | 0.94 | Income percentile |
| <0.001 | 1.30-1.46 | 1.37 | PH |

PH = pulmonary hypertension; CHF = congestive heart failure; CKD = chronic kidney disease; CPD = chronic pulmonary disease; Deyo- CCI= Deyo- Charlson Comorbidity Index; PVD = peripheral vascular disease; OSA = obstructive sleep apnea; RV = right ventricle; MI = myocardial infarction; AF/AFL= atrial fibrillation/flutter; VF/VT= ventricular tachycardia/ fibrillation; ICD = implantable cardioverter-defibrillator; PPM= permanent pacemaker.

Supplemental Table 6: Predictors for post procedural respiratory failure

| p value | **95 CI** | OR | **Correlates** |
| --- | --- | --- | --- |
| <0.001 | 0.97-0.98 | 0.97 | Age |
| 0.003 | 0.86-0.97 | 0.92 | Female |
| 0.08 | 0.87-1.01 | 0.93 | Hypertension |
| <0.001 | 1.17-1.37 | 1.27 | CHF |
| <0.001 | 0.68-0.77 | 0.72 | Diabetes mellitus |
| 0.744 | 0.92-1.06 | 0.99 | CKD |
| 0.001 | 1.05-1.21 | 1.13 | CPD |
| 0.001 | 1.05-1.21 | 1.13 | Obesity |
| <0.001 | 1.68-1.95 | 1.81 | PVD |
| 0.212 | 0.97-1.14 | 1.05 | OSA |
| <0.001 | 0.43-0.59 | 0.50 | Syncope |
| <0.001 | 2.34-2.76 | 2.54 | Cardiogenic shock |
| 0.454 | 0.64-1.22 | 0.89 | RV failure |
| <0.001 | 0.67-0.80 | 0.73 | MI |
| <0.001 | 1.48-1.67 | 1.58 | AF/AFL |
| <0.001 | 1.14-1.34 | 1.24 | VT/VF |
| <0.001 | 0.67-0.77 | 0.72 | Disorder in prior ECG |
| <0.001 | 1.88-2.16 | 2.01 | Complete AV block |
| <0.001 | 0.71-0.83 | 0.76 | Sick sunus syndrome |
| <0.001 | 1.25-1.52 | 1.38 | Cardiac arrest |
| <0.001 | 1.14-1.34 | 1.24 | Temporary pacemaker |
| 0.751 | 0.89-1.07 | 0.97 | CRT |
| <0.001 | 0.59-0.82 | 0.69 | ICD |
| 0.7 | 0.87-1.23 | 1.04 | PPM |
| <0.001 | 1.42-1.59 | 1.50 | Deyo- CCI |
| <0.001 | 1.10-1.23 | 1.16 | Hospital region |
| 0.52 | 0.95-1.00 | 0.97 | Income percentile |
| <0.001 | 0.68-0.78 | 0.73 | History of smoking |
| <0.001 | 1.15-1.32 | 1.23 | Teaching hospital |
| <0.001 | 1.34-1.56 | 1.44 | PH |

PH = pulmonary hypertension; CHF = congestive heart failure; CKD = chronic kidney disease; CPD = chronic pulmonary disease; Deyo- CCI= Deyo- Charlson Comorbidity Index; PVD = peripheral vascular disease; OSA = obstructive sleep apnea; RV = right ventricle; MI = myocardial infarction; AF/AFL= atrial fibrillation/flutter; VF/VT= ventricular fibrillation/tachycardia; CRT= cardiac resynchronization therapy; ICD = implantable cardioverter-defibrillators ; PPM= permanent pacemaker.

Supplemental Table 7: Predictors for chest tube insertion

| p value | **95 CI** | OR | **Correlates** |
| --- | --- | --- | --- |
| <0.001 | 0.99-0.99 | 0.99 | Age |
| <0.001 | 1.19-1.26 | 1.22 | Female |
| <0.001 | 1.14-1.22 | 1.18 | White ethnicity |
| <0.001 | 0.77-0.83 | 0.79 | Hypertension |
| <0.001 | 1.36-1.47 | 1.41 | CHF |
| <0.001 | 0.79-0.84 | 0.81 | Diabetes mellitus |
| 0.844 | 0.97-1.04 | 1.00 | CKD |
| <0.001 | 1.25-1.34 | 1.29 | CPD |
| <0.001 | 0.59-0.65 | 0.62 | Obesity |
| 0.008 | 1.02-1.11 | 1.06 | PVD |
| <0.001 | 0.68-0.75 | 0.72 | OSA |
| <0.001 | 0.54-0.62 | 0.58 | Syncope |
| <0.001 | 2.23-2.44 | 2.33 | Cardiogenic shock |
| <0.001 | 1.30-1.72 | 1.49 | RV failure |
| <0.001 | 0.74-0.81 | 0.77 | MI |
| <0.001 | 1.31-1.38 | 1.35 | AF/AFL |
| 0.685 | 0.95-1.03 | 0.99 | VT/VF |
| <0.001 | 0.75-0.80 | 0.77 | Disorder in prior ECG |
| <0.001 | 1.43-1.58 | 1.50 | Cardiac arrest |
| <0.001 | 1.07-1.16 | 1.11 | Temporary pacemaker |
| <0.001 | 1.16-1.27 | 1.22 | CRT |
| <0.001 | 1.28-1.39 | 1.33 | PPM |
| <0.001 | 1.26-1.34 | 1.30 | Deyo- CCI |
| <0.001 | 1.20-1.27 | 1.23 | Hospital region |
| 0.18 | 0.99-1.02 | 1.01 | Income percentile |
| <0.001 | 0.86-0.91 | 0.88 | History of smoking |
| 0.002 | 1.07-1.33 | 1.19 | Alcohol dependency |
| <0.001 | 1.11-1.19 | 1.15 | Teaching hospital |
| <0.001 | 1.32-1.42 | 1.37 | PH |

PH = pulmonary hypertension; CHF = congestive heart failure; CKD = chronic kidney disease; CPD = chronic pulmonary disease; Deyo- CCI= Deyo- Charlson Comorbidity Index; PVD = peripheral vascular disease; OSA = obstructive sleep apnea; RV = right ventricle; MI = myocardial infarction; AF/AFL= atrial fibrillation/flutter; VF/VT= ventricular fibrillation/tachycardia; CRT= cardiac resynchronization therapy; PPM= permanent pacemaker.

Supplemental Table 8: Predictors for pleural effusion

| p value | **95 CI** | OR | **Correlates** |
| --- | --- | --- | --- |
| <0.001 | 0.99-0.99 | 0. 99 | Age |
| <0.001 | 1.13-1.19 | 1.16 | Female |
| <0.001 | 1.10-1.17 | 1.14 | White ethnicity |
| <0.001 | 0.85-0.99 | 0.88 | Hypertension |
| <0.001 | 1.09-1.17 | 1.13 | CHF |
| <0.001 | 0.73-0.78 | 0.76 | Diabetes mellitus |
| 0.814 | 0.96-1.03 | 0.99 | CKD |
| <0.001 | 1.11-1.18 | 1.14 | CPD |
| <0.001 | 0.64-0.69 | 0.67 | Obesity |
| <0.001 | 1.14-1.24 | 1.19 | PVD |
| <0.001 | 0.76-0.83 | 0.79 | OSA |
| <0.001 | 0.58-0.66 | 0.62 | Syncope |
| <0.001 | 1.88-2.06 | 1.97 | Cardiogenic shock |
| <0.001 | 1.33-1.73 | 1.52 | RV failure |
| <0.001 | 0.65-0.71 | 0.68 | MI |
| <0.001 | 1.62-1.71 | 1.66 | AF/AFT |
| <0.001 | 1.10-1.19 | 1.14 | VT/VF |
| <0.001 | 0.75-0.80 | 0.78 | Disorder in prior ECG |
| <0.001 | 1.13-1.21 | 1.17 | Complete AV block |
| <0.001 | 0.80-0.86 | 0.83 | Sick sunus syndrome |
| <0.001 | 1.49-1.64 | 1.56 | Cardiac arrest |
| <0.001 | 1.28-1.39 | 1.33 | Temporary pacemaker |
| 0.001 | 1.03-1.11 | 1.67 | CRT |
| <0.001 | 0.69-0.75 | 0.72 | ICD |
| <0.001 | 1.5-1.59 | 1.55 | Deyo- CCI |
| <0.001 | 1.11-1.17 | 1.14 | Hospital region |
| <0.001 | 0.76-0.81 | 0.78 | History of smoking |
| 0.07 | 0.99-1.23 | 1.10 | Alcohol dependency |
| <0.001 | 1.14-1.22 | 1.18 | Teaching hospital |
| <0.001 | 1.48-1.59 | 1.53 | PH |

PH = pulmonary hypertension; CHF = congestive heart failure; CKD = chronic kidney disease; CPD = chronic pulmonary disease; Deyo- CCI= Deyo- Charlson Comorbidity Index; PVD = peripheral vascular disease; OSA = obstructive sleep apnea; RV = right ventricle; MI = myocardial infarction; AF/AFL= atrial fibrillation/flutter; VF/VT= ventricular fibrillation/tachycardia; CRT= cardiac resynchronization therapy; ICD = implantable cardioverter-defibrillators.

Supplemental Table 9: Predictors for pneumo\hemothorax

| p value | **95 CI** | OR | **Correlates** |
| --- | --- | --- | --- |
| <0.001 | 1.00-1.01 | 1.01 | Age |
| <0.001 | 1.43-1.54 | 1.48 | Female |
| <0.001 | 0.13-1.23 | 1.18 | White ethnicity |
| <0.001 | 0.82-0.90 | 0.86 | Hypertension |
| <0.001 | 0.78-0.86 | 0.82 | CHF |
| <0.001 | 0.55-0.60 | 0.57 | Diabetes mellitus |
| <0.001 | 0.76-0.83 | 0.79 | CKD |
| <0.001 | 1.18-1.29 | 1.24 | CPD |
| <0.001 | 0.45-0.51 | 0.48 | Obesity |
| 0.738 | 0.94-1.05 | 0.99 | PVD |
| <0.001 | 0.71-0.80 | 0.75 | OSA |
| <0.001 | 0.65-0.75 | 0.70 | Syncope |
| <0.001 | 1.62-1.86 | 1.73 | Cardiogenic shock |
| <0.001 | 0.79-0.88 | 0.83 | MI |
| 0.469 | 0.98-1.05 | 1.01 | AF/AFL |
| 0.02 | 0.88-0.99 | 0.94 | VT/VF |
| <0.001 | 0.87-0.94 | 0.91 | Disorder in prior ECG |
| 0.016 | 1.01-1.10 | 1.05 | Complete AV block |
| <0.001 | 1.07-1.16 | 1.11 | Sick sunus syndrome |
| <0.001 | 1.19-1.36 | 1.27 | Cardiac arrest |
| 0.02 | 0.86-0.97 | 0.91 | Temporary pacemaker |
| <0.001 | 1.15-1.30 | 1.22 | CRT |
| 0.152 | 0.82-1.03 | 0.92 | ICD |
| 0.577 | 0.86-1.09 | 0.97 | PPM |
| <0.001 | 1.16-1.23 | 1.19 | Deyo- CCI |
| <0.001 | 1.17-1.25 | 1.21 | Hospital region- south/west |
| 0.013 | 1.00-1.04 | 1.02 | Income percentile |
| 0.90 | 0.96-1.04 | 0.99 | History of smoking |
| <0.001 | 1.06-1.18 | 1.12 | PH |

PH = pulmonary hypertension; CHF = congestive heart failure; CKD = chronic kidney disease; CPD = chronic pulmonary disease; Deyo- CCI= Deyo- Charlson Comorbidity Index; PVD = peripheral vascular disease; OSA = obstructive sleep apnea; MI = myocardial infarction; AF/AFL= atrial fibrillation/flutter; VF/VT= ventricular fibrillation/tachycardia; CRT= cardiac resynchronization therapy; ICD = implantable cardioverter-defibrillators ; PPM= permanent pacemaker.

Supplemental Table 10: Predictors for pocket hematoma

| p value | **95 CI** | OR | **Correlates** |
| --- | --- | --- | --- |
| <0.001 | 0.98-0.99 | 0. 99 | Age |
| <0.001 | 1.06-1.22 | 1.13 | Female |
| 0.192 | 0.97-1.15 | 1.06 | White ethnicity |
| 0.117 | 0.85-1.02 | 0.93 | Hypertension |
| <0.001 | 1.38-1.67 | 1.52 | CHF |
| <0.001 | 0.80-0.94 | 0.87 | Diabetes mellitus |
| <0.001 | 1.12-1.33 | 1.22 | CKD |
| <0.001 | 0.633-0.77 | 0.70 | CPD |
| <0.001 | 0.67-0.81 | 0.74 | Obesity |
| <0.001 | 1.16-1.45 | 1.29 | PVD |
| 0.001 | 0.75-0.93 | 0.83 | OSA |
| <0.001 | 0,52-0.72 | 0.62 | Syncope |
| 0.31 | 1.01-1.34 | 1.17 | Cardiogenic shock |
| 0.294 | 0.86-1.05 | 0.95 | MI |
| <0.001 | 1.22-1.41 | 1.31 | AF/AFL |
| <0.001 | 0.70-0.86 | 0.78 | VT/VF |
| <0.001 | 0.60-0.71 | 0.65 | Disorder in prior ECG |
| 0.401 | 0.95-1.13 | 1.04 | Complete AV block |
| <0.001 | 0.45-0.54 | 0.49 | Sick sunus syndrome |
| <0.001 | 0.64-0.88 | 0.75 | Cardiac arrest |
| <0.001 | 1.15-1.42 | 1.27 | CRT |
| <0.001 | 0.35-0.48 | 0.41 | ICD |
| <0.001 | 0.42-0.58 | 0.49 | PPM |
| 0.873 | 0.94-1.08 | 1.01 | Deyo- CCI |
| <0.001 | 0.80-0.91 | 0.85 | Hospital region- south/west |
| 0.073 | 0.99-1.06 | 1.03 | Income percentile |
| <0.001 | 0.81-0.94 | 0.87 | History of smoking |
| 0.018 | 1.02-1.25 | 1.13 | PH |

PH = pulmonary hypertension; CHF = congestive heart failure; CKD = chronic kidney disease; CPD = chronic pulmonary disease; Deyo- CCI= Deyo- Charlson Comorbidity Index; PVD = peripheral vascular disease; OSA = obstructive sleep apnea; MI = myocardial infarction; AF/AFL= atrial fibrillation/flutter; VF/VT= ventricular fibrillation/tachycardia; CRT= cardiac resynchronization therapy; ICD = implantable cardioverter-defibrillators ; PPM= permanent pacemaker.

Supplemental Table 11: Predictors for any in hospital complications

| p value | **95 CI** | OR | **Correlates** |
| --- | --- | --- | --- |
| <0.001 | 0.99-0.99 | 0. 99 | Age |
| <0.001 | 1.15-1.19 | 1.17 | Female |
| <0.001 | 0.84-0.88 | 0.86 | Hypertension |
| <0.001 | 1.09-1.14 | 1.11 | CHF |
| <0.001 | 0.74-0.77 | 0.75 | Diabetes mellitus |
| 0.141 | 0.97-1.01 | 0.99 | CKD |
| <0.001 | 1.09-1.14 | 1.12 | CPD |
| <0.001 | 0.78-0.82 | 0.80 | Obesity |
| <0.001 | 1.12-1.17 | 1.14 | PVD |
| <0.001 | 0.82-0.87 | 0.84 | OSA |
| <0.001 | 0.61-0.66 | 0.64 | Syncope |
| <0.001 | 2.62-2.77 | 2.70 | Cardiogenic shock |
| <0.001 | 1.33-1.46 | 1.46 | RV failure |
| <0.001 | 0.77-0.81 | 0.79 | MI |
| <0.001 | 1.26-1.31 | 1.29 | AF/AFL |
| <0.001 | 1.14-1.19 | 1.16 | VT/VF |
| <0.001 | 0.79-0.82 | 0.80 | Disorder in prior ECG |
| <0.001 | 1.09-1.14 | 1.12 | Complete AV block |
| <0.001 | 0.84-0.87 | 0.85 | Sick sunus syndrome |
| <0.001 | 1.69-1.78 | 1.73 | Cardiac arrest |
| <0.001 | 1.34-1.40 | 1.37 | Temporary pacemaker |
| <0.001 | 1.14-1.20 | 1.17 | CRT |
| <0.001 | 0.66-0.73 | 0.69 | ICD |
| 0.169 | 0.92-1.02 | 0.97 | PPM |
| <0.001 | 1.3-1.34 | 1.32 | Deyo- CCI |
| <0.001 | 1.03-1.07 | 1.05 | Hospital region- south/west |
| <0.001 | 0.82-0.85 | 0.84 | History of smoking |
| 0.127 | 0.61-0.66 | 0.95 | Alcohol dependency |
| <0.001 | 1.22-1.27 | 1.24 | PH |

PH = pulmonary hypertension; CHF = congestive heart failure; CKD = chronic kidney disease; CPD = chronic pulmonary disease; Deyo- CCI= Deyo- Charlson Comorbidity Index; PVD = peripheral vascular disease; OSA = obstructive sleep apnea; RV= right ventricle; MI = myocardial infarction; AF/AFL= atrial fibrillation/flutter; VF_VT= ventricular fibrillation/tachycardia; CRT= cardiac resynchronization therapy; ICD = implantable cardioverter-defibrillators ; PPM= permanent pacemaker.
